# High Emigration Propensity and Low Mortality on Transfer Drives Female-Biased Dispersal of *Pyriglena leucoptera* in Fragmented Landscapes

**DOI:** 10.1371/journal.pone.0170493

**Published:** 2017-01-20

**Authors:** Marcelo Awade, Carlos Candia-Gallardo, Cintia Cornelius, Jean Paul Metzger

**Affiliations:** 1 Department of Ecology, Institute of Biosciences, University of São Paulo (USP), São Paulo, SP, Brazil; 2 Department of Biology, Institute of Biological Sciences, Federal University of Amazonas (UFAM), Manaus, AM, Brazil; University of Maine, UNITED STATES

## Abstract

Dispersal is a biological process performed in three stages: emigration, transfer and immigration. Intra-specific variation on dispersal behavior, such as sex-bias, is very common in nature, particularly in birds and mammals. However, dispersal is difficult to measure in the field and many hypotheses concerning the causes of sex-biased dispersal remain without empirical confirmation. An important limitation of most empirical studies is that inferences about sex-biased dispersal are based only on emigration proneness or immigration success data. Thus, we still do not know whether sex-biased immigration in fragmented landscapes occurs during emigration, transfer or in both stages. We conducted translocation and radiotracking experiments to assess i) whether inter-patch dispersal movements of a rainforest bird (*Pyriglena leucoptera*) is sex-biased and ii) how dispersal stages and the perceptual range of the individuals are integrated to generate dispersal patterns. Our results showed that inter-patch dispersal is sex-biased at all stages for *P. leucoptera*, as females not only exhibit a higher emigration propensity but are subjected to a lower risk of predation when moving through the matrix. Moreover, our data support a perceptual range of 80 m and our results showed that dispersal success decreases considerably when inter-patch distances exceeds this perceptual range. In this case, birds have a higher probability of travelling over longer routes and, as a consequence, the risk of predation increases, specially for males. Overall, results supported that assuming dispersal as a single-stage process to describe dispersal behavior may be misleading. In this way, our study advanced our understanding of processes and patterns related to inter-patch dispersal of neotropical forest birds, shedding light on potential implications for population dynamics and for the management of fragmented landscapes.

## Introduction

Dispersal is a key concept in ecology and evolution and, probably, the most studied movement process in literature [1, 2]. This process is defined as a change of the geographical location of the home range of an individual or, simply, as a movement away from an individual’s home range with no return [3]. Dispersal may have profound effects on population demography and genetics, not only by altering the number of individuals, sex ratio and age structure of a population, but also by allowing gene flow among populations [3]. Despite the ubiquitousness of dispersal in ecological and evolutionary theory, an integrated and synthetic understanding of the causes and consequences of dispersal has been achieved just recently [1]. Actually, this process is very difficult to study empirically and dispersal research has been based on simplistic assumptions [1, 2, 4, 5].

Under an evolutionary perspective, dispersal is often assumed to be an unconditional process (i.e. out of the individuals’ control; an innate process) and is considered a selected behavior which increases fitness of individuals through kin competition reduction and inbreeding avoidance [6, 7]. As a result, much theory was focused on determining the ultimate causes of dispersal [1, 2] and theoretical models derived from this background usually assume that all costs of dispersal are paid at emigration and these costs can be encapsulated into a single constant (*i.e.* an evolutionary stable dispersal rate [8]). This conception of dispersal has, at least, two limitations that have been hardly questioned in the recent literature. First, dispersal should be conceived as a process occurring in three stages: emigration, transfer and immigration [1]. This issue was raised almost 20 years ago [9], but studies adopting this definition of dispersal are very scarce. Second, dispersal is a result of long-term evolutionary processes and also determined by ongoing natural selection process shaping individual short-term decisions in response to environmental conditions and constraining both motion and navigation capacities of individuals [10]. For example, there is a growing theoretical and empirical literature demonstrating that spatial processes (e.g. habitat destruction and fragmentation) may affect the dispersal behavior of an individual [1, 2].

Habitat destruction and fragmentation affects landscape configuration by decreasing the size and increasing the isolation of habitat patches [11]. Patch isolation effects, for instance, are related to the costs of dispersal in fragmented landscapes. Energetic costs increase with the time an individual expends travelling, which, in turn, increase with inter-patch distance [4]. Predation risk is also expected to increase when an animal moves through the matrix [12], even though predation events are very difficult to be observed empirically. In addition, perceptual range plays an important role by affecting not only the emigration propensity of an individual, but also the transfer and immigration success. On one hand, perceptual range limitations may decrease the chance of finding another patch in the landscape [13]. Consequently, individuals that choose to emigrate have a greater chance of leaving a patch in a direction different than that of the nearest patch when the inter-patch distance is larger than their perceptual range. In this case, we should expect not only an increase in the cost of moving compared to the least costly path but also an increase in the risk of predation in the matrix [14, 15]. On the other hand, animals may change their movement behavior from a random walk like to a more straight correlated random walk when they are able to detect a neighbor patch [16]. This behavioral change is expected to increase survival during transfer and immigration success [15].

Another commonly neglected aspect in dispersal theory, particularly in population ecology, is intra-specific variation on dispersal behavior. Sex-biased dispersal, for example, is a pattern that has been described early in dispersal theory [7, 17]. This pattern is commonly studied under the framework of Greenwood’s hypothesis [17], stating that in socially monogamous species, which seems to be a widespread pattern in birds, males compete for resources and tend to be philopatric, whereas females disperse looking for mates. On the other hand, in polyginous species, which is the case of most mammals, males compete for mates, making females more philopatric to take alone the costs of offspring’s care. Thus, dispersal is expected to be male-biased in mammals and female-biased in birds, but a number of exceptions to this pattern have been found (*e.g.* [18, 19]).

Proximate causes should also play a key role on determining sex-biased dispersal, as males and females may show distinct short-term behaviors in response to demographic parameters or to spatial pattern. Empirical studies analysing sex-biased dispersal are strongly based on immigration success data, which may be considered a shortcoming [1]. Sex-biased immigration may be a consequence of biased emigration, biased mortality risk when moving through the matrix or a combination of both. To our knowledge, there are no macroscale studies that have integrated dispersal stages when investigating sex-biased dispersal.

This lack of studies can be partially explained by practical constrains, since tracking individual movements in the field is difficult and sometimes impossible. Animal movements are hard to follow and usually require a large effort to monitor a small number of individuals [3]. In the case of dispersal, detecting the exact moment when a dispersal event occurs is very unlikely. Mark-recapture and molecular techniques are helping to find some patterns, but only provide indirect evidence of the process [2]. The movement itself and its behavioral features are not evaluated by these techniques, precluding a mechanistic understanding of the phenomenon. These shortcomings have been overcome by the recent advances in telemetry technology [20, 21] and by the use of translocation experiments [22, 23].

Here, we conducted a translocation and radiotracking experiment to assess whether inter-patch dispersal is sex biased for a tropical rainforest bird (White-shouldered Fire-eye, *Pyriglena leucoptera*, Vieillot, 1818). Moreover, our experimental design allowed us to shed light on how dispersal stages are integrated in order to produce a sex-biased pattern (i.e., if inter-patch dispersal is, in fact, sex biased). Following the abovementioned theory, our main hypotheses were that inter-patch movement success is female-biased and that this pattern is a consequence of higher emigration propensity and lower or equal movement costs and mortality risk when moving through the matrix for females. Therefore, we predict that females i) emigrate more and take less time to leave a small forest patch than males, ii) spend equally or less time and iii) are equally or less predated in the matrix habitat when moving and, consequently, iv) have more success on arriving in a neighbor forest patch. Together with the main hypotheses, we tested the existence of a perceptual range for the species. We predicted that there is an inter-patch distance above which the movement pattern of an individual changes from directed to the nearest neighbour to a random direction in the landscape. Understanding these dispersal patterns contributes to advance our knowledge of the causes of sex-biased dispersal and provide us important information to be used on theoretical modeling and on planning appropriate management practices for the conservation of forest bird species in fragmented landscapes.

## Methods

### Study area

This study was conducted in a region of the Atlantic Plateau of São Paulo (47°20’ W - 48°40’ W and 23°43’ S - 24°06’ S; S1 Fig), a highland region in the Atlantic Forest domain that runs almost parallel to the coastline in the east-southeast of Brazil. The climate type in this area is subtropical and rainy (Cwa sensu Köppen) and the vegetation is dominated by lower montane rainforests [24] but can be strongly influenced by semi-deciduous forests in some areas [25]. The study area is a fragmented landscape dominated by small habitat patches of secondary forest (< 100 ha; S1 Fig). Forest cover in the study area ranges from 11 to 49%, considering 10,000 ha landscape samples. Forest patches are inserted in a matrix dominated by croplands and pastures (> 80% of the matrix), but other land uses, such as dense shrublands, exotic pine and *Eucalyptus* sp. plantations, are also found. The fragmented part of the study area is adjacent to a very large patch (> 1,000,000 ha of continuous forest; [26].

### Study species

*Pyriglena leucoptera* (White-shouldered Fire-eye, Thamnophillidae) is 30 g insectivorous bird endemic of the Atlantic Forest. It is usually found in the understory of primary and secondary forests [27, 28]. This species was chosen since it has a marked sexual dimorphism [27] and because it is one of few forest bird species for which ecological knowledge is available in the literature. In the Atlantic Plateau of São Paulo, this species is fairly common in fragmented landscapes where forest cover exceeds 30%, but uncommon where less than 15% forest cover remains [29, 30]. Its territory size is estimated to be less than 2 ha [31] based on color banding and spot-mapping analyses, and its mean home range size is estimated at up to 15.4 ha based on radiotelemetry data [32]. This discrepancy may be partially explained by sporadic extra-territorial movements that individuals do when following army ants [33].

### Experimental design

We followed the translocation-and-radiotracking experimental design proposed by [34] with small modifications. The main distinction of our approach in relation to Castellón and Sieving’s study [34] was that we evaluated the effects of patch isolation on dispersal behavior instead of comparing the permeability of three matrix types. Birds were captured in continuous forests sites (> 1,000,000 ha) and translocated to very small forest patches far away from capture sites to avoid homing behavior [23, 35]. The basic reasoning of this kind of translocation experiments is to simulate a situation analogous to that of an individual dispersing when the habitat is fragmented [34]. We controlled release sites characteristics, as much as possible, in order to provide a standardized dispersal stimulus for all individuals.

#### Capture sites, bird capture and transmitter attachment

We captured adult *P. leucoptera* individuals with mist nets and banded them with unique numbered metal bands. Then, birds were radio tagged following the glue on attachment procedure. This technique consists in remove the feathers from the back of the birds and glued the transmitter on their backs with a latex glue (see [36] and [37] for details). We made all efforts to minimize stress on animals during the attachment procedure. Radiotransmitters were produced by Telenax and we used the model TXC-001G. They weigth 0.6 g, the battery had a mean lifetime of 14 days (according to Telenax) and the range of frequencies were from 148 to 174 MHz. The glue on is probably the less invasive attachment technique [38] and transmitters detached from animals in less than 30 days.

We tagged and translocated only adult individuals as juveniles may have different motivations to move and were captured in smaller numbers than adults. We avoided capturing birds during the peak of the breeding season (September to December) in order to avoid an impact on the reproduction of individuals. We conducted three capture sessions: i) from January to March of 2008, ii) from May to August of 2008 and iii) on February of 2009. Capture sites were at the interior of the continuous forests (> 2.5 km from the forest edge) and were located more than 10 km from release sites. Thus, we translocated birds that supposedly had no previous experience with disturbed habitats. Individuals were kept safe in opaque fabric bags in order to reduce stress during transportation from capture sites to release sites. Since no surgical procedure were performed with birds, no ethical permission from any specific ethical committee was required to conduct this research. All permissions to capture, handling, banding, translocating and to glue the transmitters on birds were provided by Brazilian Institute of the Environment (IBAMA), through the licenses IBAMA/SISBio n. 12426-1 and IBAMA/CEMAVE n. 2959/2. We must note that SISBio and CEMAVE are now under the administration of the Chico Mendes Institute for Biodiversity Conservation (ICMBio), the Brazilian institution regulating field work research with living organisms.

#### Release sites

We translocated birds to 14 distinct release sites, consisting of a release patch, its nearest neighbor and the matrix surrounding these patches. Release patches had similar conditions in terms of climate, vegetation and relief slopes, and were small enough to satisfy only the immediate requirements of the translocated birds in terms of food and shelter [34]. The size of release patches ranged from 0.19 to 3.31 ha (mean ± sd = 1.22 ± 0.92; S1 Table). Since size variation among release patches was small, we assumed that this variation was not enough to elicit a patch area effect on the dispersal behavior of translocated individuals. In addition, the release patch area was not correlated with the nearest neighbor distance (r = 0.21, df = 12, p = 0.46) in order to avoid confounding effects between patch area and inter-patch distance. We provided an equivalent dispersal stimulus for all tested birds by standardizing the release sites as much as possible with regard to habitat quality at both patches (i.e. release and nearest neighbor patches). Moreover, we controlled for the non-occurrence of conspecifics in the release patches and we assumed that the nearest neighbor patches were sufficiently large to consider it as a suitable patch (i.e. larger than 15 ha in most cases; see S1 Table). Unfortunately, we could not control for conspecifics occurrence in the nearest neighbor patches. However, we assumed that this was not a big issue in our study, since our experimental design provided a strong stimulus for dispersal, no matter whether there were conspecifics in neighboring patches.

**Fig 1:**
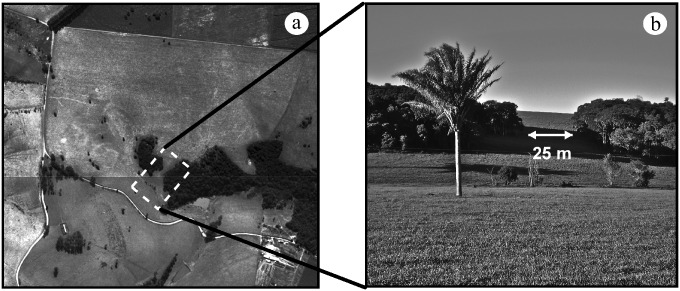
Example of a release site. A detailed aerial view (a) and a digital photography (b) showing the release site 8 (RS8). In this release site, the distance between the release patch and its nearest neighbor is 25 m. The forest fragment in the center of a) and in the left of b) was the release patch (area = 1.21 ha). See S1 Table for the spatial characteristics of release sites.

Altogether, these procedures allowed us to assume that inter-patch distance was the main environmental variable affecting dispersal decisions. To standardize gap characteristics, we included only open-type matrices (i.e. pastures or low croplands; Fig 1). The shortest distance between the release patch and its nearest neighbor (hereafter, nearest neighbor distance) was our measure of isolation. Nearest neighbor distances varied from 25 to 340 m among the release sites. All spatial metrics were calculated using ESRI ArcMap 9.2 software with the XTools and Conefor input extensions [39].

#### Sampling distribution and bird radiotracking

Our sample sizes were small hindering us to analyse the effects of seasonality. Thus, we uniformly distributed the 14 release sites throughout the study period to avoid potential seasonal confounding effects on interpretation of the results. In this way, there was no relation between the mean nearest neighbor distance and the season when a particular distance was tested (r = −0.15, df = 12, p = 0.60). We assumed that season was not an issue in our study, since we avoided sampling during the peak of the breeding period, in which birds presumably are more active. Moreover, we considered that any potential season effect is overshadowed by the effects of patch isolation and sex due to our experimental design.

Birds were individually translocated to each release patch and monitored one at a time. Individuals were systematically tracked over five consecutive days or until completion of an inter-patch movement if it occurred before this time span. Bird locations were obtained hourly except during the first two hours after release or after dawn, and during the last two hours before dusk, when locations were obtained every 15 min. After five days or after an inter-patch movement event, locations were obtained once a day whenever possible. Although we attempted to obtain a complete systematic monitoring dataset for at least one bird per sex at each release site, the transmitter of one female detached prior to the end of the systematic monitoring period. We considered the fate of these female as unknown (i.e. censored).

#### Response variables

Our radiotracking protocol enabled us to gather six response variables: emigration propensity, departure direction, time spent on transfer, predation occurrence, flight capability and inter-patch movement success. Emigration propensity was measured as the time birds spent in the release patch before emigrating. We opted to consider time in terms of daylight hours because bird activity is assumed to decrease during the night, and no birds emigrated during night hours. Departure direction is an angle formed by the segment with its origin at the center of the release patch and passing through the first location obtained outside this patch, and the segment starting at the center of the release patch and passing through the center of the nearest neighbor patch. This angle measures how far the direction of departure deviated from the direction of the nearest patch (i.e. reference or least-cost direction).

Time spent on transfer (i.e. the cost associated to moving in open matrices) was measured by the time (in hours) an individual spent in the matrix before arriving to a forest patch. Predation occurrence is a measure of mortality risk during the transfer stage and indicated whether an individual that emigrated was predated in the matrix or not. Predation events were determined directly in cases we observed the predator with its prey or indirectly when we found typical traces of predation (*i.e.* the transmitter was found with parts of the animal). The type of locomotion, hereafter referred to as flight capability, accounted for the motion capacity of the birds and indicated whether they flew or hopped during transfer. Inter-patch movement success, whether a bird reached another forest patch in the landscape or not, depends on the emigration of the individual and takes into account the route travelled in the matrix, as some birds may not arrive in the nearest neighbor patch but in another patch. Thus, inter-patch movement success synthesizes the success of the dispersal process, including the first step of the immigration stage (i.e. arriving in an habitat patch that potentially allows individual establishment, the second step). Unfortunately, we could not determine individual establishment in the arrival patch due to the small lifetime of transmitters and logistical constrains (e.g. when birds moved to land properties in which we were not authorized to work).

### Data analysis

#### Model selection

We applied a model selection approach in order to make inferences about the effects of distance and sex on emigration propensity, time spent on transfer and inter-patch movement success, as well as to estimate the species perceptual range. We built an unique set of models to explain the variation of each response variables. We used the second-order Akaike information criterion (AICc) to evaluate the plausibility of the competing models. We based our inferences on the best model of the set (the one with the lowest AICc value), even though every model with a ΔAICc < 2.0 was considered as equally plausible [40]. Remarks on these models were made whenever necessary. The strength of evidence in favor of a model being the best one was given by its Akaike weight (w_i_). All analyses were run using R 2.11 software [41] with the bbmle, survival, circular and CircStats packages.

#### Survival and generalized linear models

We used parametric survival models to analyze emigration propensity and time spent on transfer [42]. In the context of our study, survival means that an individual either stayed at the release patch (for emigration propensity analysis) or at the matrix habitat (for time spent on transfer analysis) for a time smaller or equal than *t*. In turn, a death event means that an individual emigrated or arrived in a forest patch. We modeled the survival function as a Weibull distribution, allowing us to evaluate whether the hazard rates (i.e. the instantaneous probability that an individual emigrates or arrives in a forest patch at time *t*, given that it stayed in the release patch or in the matrix habitat until *t*) are constant or vary as a function of time. Despite having only two parameters, scale (λ) and shape (ρ), the Weibull distribution is very flexible. It has the exponential distribution as a particular case (when ρ = 1), which means that the hazard rate is constant over time and equal to λ. If ρ > 1, the hazard rate increases with time, meaning that the probability of emigration or arrival in a forest patch increases as individuals stay longer in the release patches or in the matrix environment. The opposite occurs if ρ < 1, when the hazard rate decreases with time. We analyzed the 95% confidence interval (CI) of the estimated ρ to determine whether the hazard rate varies in time or not. If the 95% CI(ρ) included the value ρ = 1, we considered the hazard rate as constant over time and we modeled the survival function as an exponential distribution. We modeled λ as a function of our predictors, thus hazard rates estimates are also allowed to vary as a function of inter-patch distance and sex.

We used generalized linear models to analyze inter-patch movement success. This response variable followed a binomial distribution and we considered the logistic link function when inter-patch distance was included in the model. We would use the same procedure to analyze flight capacity and predation occurrence, however we did not have a proper sample sizes to conduct quantitative analyzes of these variables. Thus, we treated flight capability and predation occurrence only qualitatively.

We must mention that we used distinct measures of inter-patch distance depending on the response variable been analyzed. For emigration propensity models, we considered the nearest neighbor distance (NND) as our distance measure. The matrix habitat was homogeneous among all release sites, so we took the nearest neighbour direction as the least-cost one for a bird willing to disperse. For time spent on transfer and inter-patch movement success analysis, our distance measure was the length of the shortest path between the release patch and the nearest existing patch in the departure direction. This distinction was necessary because the costs change when birds do not disperse through the least-cost direction and move to a longer route before finding a suitable patch in the landscape.

The sets of models we used to analyze emigration propensity, time spent on transfer and inter-patch movement success were composed by five models with the following general structure: i) a global model, including the interactive effects of sex and distance; ii) an additive model, considering the effects of distance and sex without their interaction; iii) a distance model, in which the response variable is affected only by distance; iv) a sex model, including only the effects of sex; and v) a “null” model, in which the response variables are not affected by any of these predictors.

#### Circular models and species’ perceptual range

No prior information on the species perceptual ability is available in literature and there is no straightforward technique to measure the perceptiveness of an animal. We assumed that individuals move through the least-cost path while dispersing if they have a perception of the surrounding landscape, so the dispersal movement will be oriented to the nearest neighbor patch in this case (departure direction = reference direction = 0 rad). In the absence of perception, individuals depart from a patch in a random direction. Based on this assumption, we used circular models to analyze the departure direction of emigrating individuals and make inferences about the perceptual range of *P. leucoptera*. We must note that our small sample sizes hampered us to evaluate the effects of sex on the perceptual range, but we expect that this limitation is weak, since visual acuity is correlated with body size [43] and both sexes have the same weight in *P. leucoptera*. In this way, we built a set of three models (M1 to M3) and applied the model selection approach to evaluate the support of three hypotheses about the species perceptual range.

The model M1 refers to the hypothesis that there is no perception of the surrounding landscape, so we used a circular uniform distribution to model departure direction variation. The model M2 refers to the hypothesis that individuals have perception of the landscape, but the perceptual range is higher than the longest inter-patch distances we studied. So, birds can always detect the nearest neighbor patch in the range of nearest neighbor distances of our study. In this case, we considered departure direction as a random variable following the von Mises distribution, a gaussian analog for circular data defined by two parameters: mean direction (*θ*) and concentration (*κ*). The mean direction was fixed, *θ* = 0 rad, in order to account that birds orient their movements to the nearest neighbor patch. Thus, *κ* is the only parameter to be estimated in M2.

The model M3, in turn, refers to the hypothesis concerning the existence of a threshold distance (d_c_) below which individuals are able to detect the nearest neighbor patch, so this model accounts for the case in which the perceptual range is inside the interval of inter-patch distances we tested here. We conducted a preliminary analysis to determine d_c_ and then obtain the final structure of M3. Basically, we categorized our distance predictor using six values of d_c_ to split our original distance variable in two groups (i.e. NND below and above d_c_). This new categorical predictor, DIST, has two levels: DIST = 1 if NND ≤ d_c_ or DIST = 0 otherwise. For each categorization of DIST, we built a model in which departure direction is affected by distance. In these models, departure direction followed a von Mises distribution with *θ* = 0 rad and *κ* = *κ*_0_ + *κ*_1_DIST. An important property of the von Mises distribution is that it approaches a circular uniform distribution when *κ* = 0. Therefore, we assumed that *κ*_0_ = 0, indicating that when DIST = 0 (i.e. NND ≤ d_c_) individuals emigrate to a random direction in the landscape. We determined the best threshold distance and the final structure of M3 by applying the model selection procedure in this set of six models and using the best selected model from this set.

## Results

### Emigration propensity

**Fig 2:**
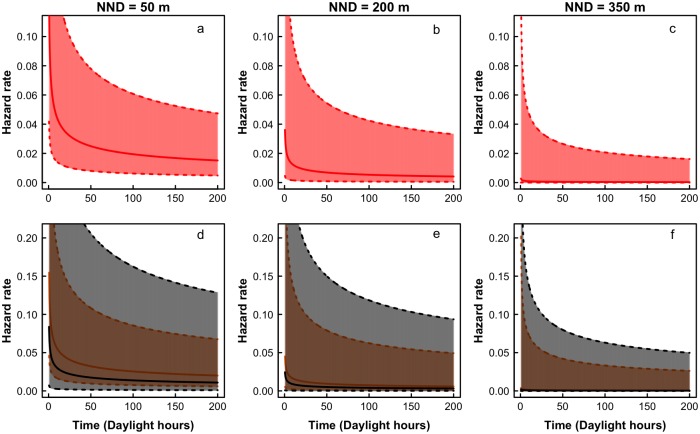
Predictions of the selected models of emigration propensity. (a-c) Variation of hazard rates over time based on the estimated parameters of the best model (emigration propensity is only affected by interpatch distance). This plot shows that the hazard rate decreases over time and as the nearest neighbor distance (NND) increases. The distance effect were presented for three instances: a) 50 m, b) 200 m and c) 350 m. 95% confidence intervals of the estimated parameters were also plotted. (d-f) Variation of hazard rates over time based on the estimated parameters of the second best model (interpatch distance and sex affect emigration propensity). This model had an equal plausibility compared to the best model. In this plot, distances are represented in different panels: d) 50 m, e) 200 m and f) 350 m. Sex is represented by different colors (black = male and orange = female). 95% confidence intervals of the estimated parameters were also plotted. We observed that the hazard rate decrease over time, as NND increases and is lower for males than females for any NND.

We translocated 31 birds (15 males and 16 females), releasing at least one bird of each sex in each experimental landscape S1 Table. We released 18 birds (nine males and nine females) in the wet season and 13 individuals (six males and seven females) in the dry season. Five out of 31 birds stayed more than five days (almost 60 daylight hours) in the release patch. The best model to explain emigration propensity was the model including only distance as a predictor (AICc = 188.79, w_i_ = 0.627). The evidence in favor of this model was very strong compared with the null model (ΔAICc = 7.63, w_i_ = 0.014). However, the model considering additive effects of distance and sex had a ΔAICc = 1.75 (w_i_ = 0.261) and should be viewed as equally plausible compared to the best model (Table 1). Together, the strength of evidence in favour of these two models sums 0.888 (≈ 0.9). All other models had very weak support (ΔAICc ≤ 3.91, w_i_ ≤ 0.089; Table 1). Considering the estimated parameters of the best model (β_Intercept_ = −1.87, SE = 0.54; β_DIST_ = −0.013, SE = 0.005), inter-patch distance had a negative effect on the proportion of individuals emigrating from release patches. A similar pattern was observed when additive effects of sex and distance were taken into account. However hazard rates were lower for males at any time interval or distance, indicating that emigration propensity was higher for females than for males (β_Intercept_ = −1.62, SE = 0.58; β_DIST_ = −0.012, SE = 0.005; β_SEX(males)_ = −0.61, SE = 0.64). In fact, four males and only one female did not leave the release patch during the monitoring period. In addition, there was no evidence supporting constant hazard rates since selected models had ρ < 1 and the 95% CI for this parameter did not include the value ρ = 1 (0.46 ≤ ρ_best_ ≤ 0.89 and 0.47 ≤ ρ_2nd best_ ≤ 0.92). This result indicates that hazard rates decreased as individuals stayed longer in the release patch (Fig 2).

**Table 1:** Model selection results for the effects of the nearest neighbor distance (NND) and sex on emigration propensity. Parametric survival models were used in these analyses.

Rank	Model Description	- LL	k	AICc	ΔAICc	w	ρ (95% CI)
1	NND	90.950	3	188.79	0	0.627	0.64 (0.46 − 0.89)
2	NND + sex	90.501	4	190.54	1.75	0.261	0.66 (0.47 − 0.92)
3	NND × sex	90.148	5	192.70	3.91	0.089	0.66 (0.48 − 0.92)
4	Null	95.998	2	196.42	7.63	0.014	0.55 (0.39 − 0.75)
5	sex	95.171	3	197.23	8.44	0.009	0.57 (0.41 − 0.80)

– LL, negative log-likelihood; k, number of parameters; AICc, second order Akaike information criterion; ΔAICc, AICc differences; w, Akaike weight; ρ, shape parameter of Weibull distribution.

### Departure direction and perceptual range

**Fig 3:**
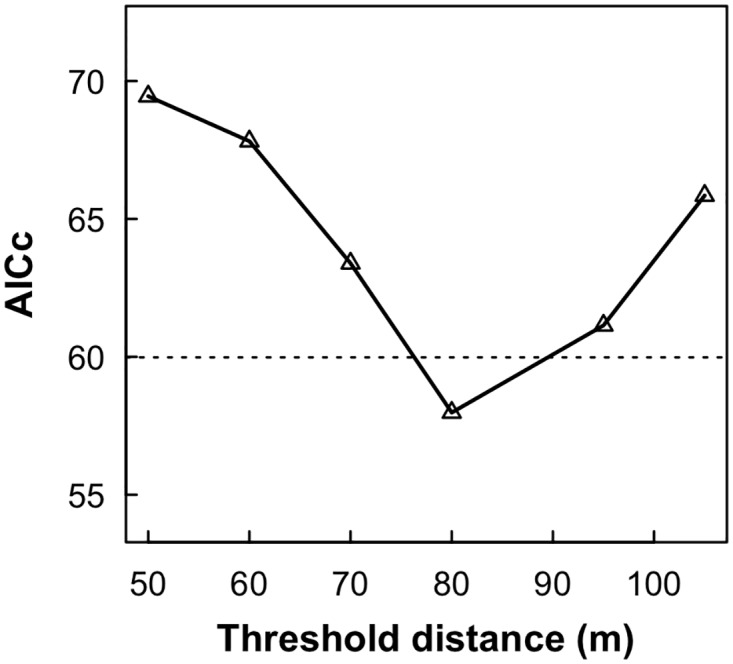
AICc profile to determine the threshold distance (d_c_) used in the categorization of the distance variable (DIST). Variation of the AICc according to d_c_. This information allowed us to define the best d_c_ according to our dataset and, in turn, the potential perceptual range of *P. leucoptera*’s individuals assuming the hypothesis in which the species’ perceptual range is inside the range of tested inter-patch distances. The dashed horizontal line indicates the standard criterium to decide which models were plausible according to data. Points above the dashed line represent models that had a ΔAICc > 2 and for which our dataset provide poor evidence to select them as plausible models. The only selected model was the one accounting for a perceptual range of 80 m.

**Fig 4:**
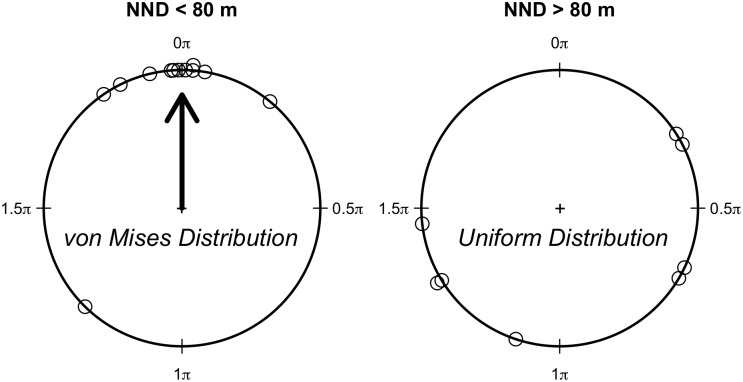
Departure direction in relation to inter-patch distance and evidence for a perceptual range. When the distance to the nearest neighbour was short (< 80 m), individuals were able to orient their movement to the nearest patch. Length of arrow indicates the magnitude birds concentrated the direction of their movements arround the reference direction (0 rad). On the other hand, when the distance to the nearest neighbour was higher than 80 m, departure direction was random. We considered this pattern as an evidence of a perceptual range around 80 m for *P. leucoptera* individuals.

We obtained the departure direction for 20 individuals (nmales = nfemales = 10). Nine of these birds (4 females and 5 males) did not leave the release patch in the direction of the nearest neighbor patch. Our preliminary analysis showed that the best threshold distance was d_c_ = 80 m (Fig 3). The best model predicting departure direction was M3 (AICc = 57.98, w_i_ = 0.997; Fig 4; Table 2), in which departure direction is random for NND > 80 m (*κ* = 0) and oriented to the nearest patch for NND ≤ 80 m (*κ* = 2.98). This model may be considered alone as the best model since the other models had an ΔAICc ≥ 11.63 and w_i_ ≤ 0.003 (Table 2). This is a strong evidence supporting a perceptual range of 80 m for *P. leucoptera*.

**Table 2:** Model selection results for the effects of nearest neighbor distance on departure direction. Circular models were used in this analysis and a detailed explanation of each model are presented on Table 1.

Rank	Model Description	- LL	k	AICc	ΔAICc	w
1	M3 (Perceptual range inside)	26.64	2	57.98	0	0.997
2	M2 (Perceptual range outside)	33.70	1	69.61	11.63	0.003
3	M1 (Null)	36.76	0	73.52	15.54	< 0.001

– LL, negative log-likelihood; k, number of parameters; AICc, second order Akaike information criterion; ΔAICc, AICc differences; w, Akaike weight.

### Time spent on transfer, flight capability and predation occurrence

**Fig 5:**
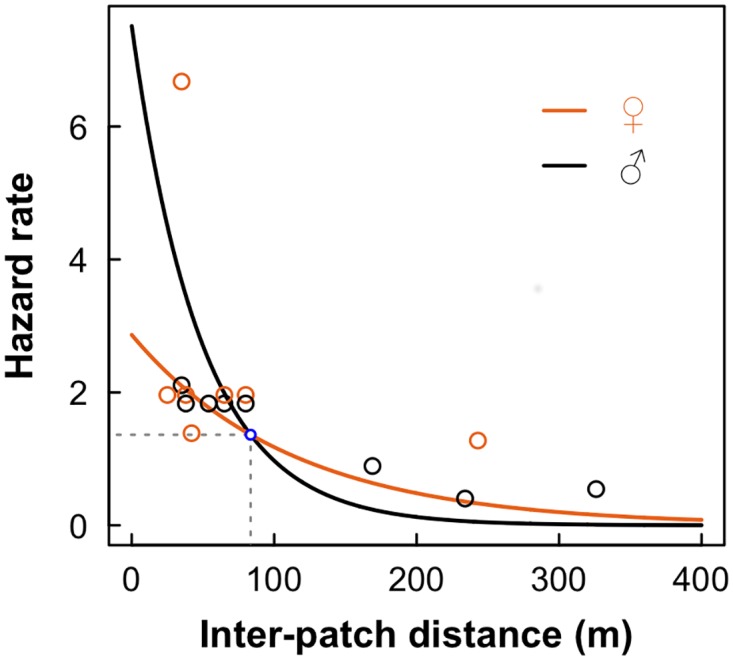
Time spent on transfer for males and females. Variation of hazard rates in relation to inter-patch distance for males (black curve and open circles) and females (orange curve and open circles). In this plot, we used the estimated parameters of the model of interactive effects between sex and distance, considering a constant hazard rate over time (ρ = 1). The point at which the hazard rate curves of males and females crosses (blue open circle) indicates the distance above which hazard rates of males become lower than that of females. This point suggests that time spent on transfer increases considerably higher for males than females, if patches are isolated by more than 86 m (a distance above the estimated perceptual range of the species).

Complete information on movement in the matrix was obtained for 18 individuals (10 males and 8 females). The best model to explain time spent on transfer was the model considering an interaction effect between distance and sex (AICc = 40.31, w_i_ = 0.495). The evidence in favor of this model was very strong compared with the null model (ΔAICc = 24.57, w_i_ < 0.001). However, this evidence was not sufficiently strong to support the interaction model alone as the best model (Table 3). The model considering only distance effects had a ΔAICc = 0.37 and a w_i_ = 0.411, and should be viewed as equally plausible compared to the best model. Considering the estimated parameters of the best model (β_Intercept_ = 1.05, SE = 0.32; β_DIST_ = −0.009, SE = 0.002; β_SEX(males)_ = 0.96, SE = 0.52; β_DIST*SEX(males)_ = −0.012, SE = 0.004), inter-patch distance had a negative effect on the proportion of individuals arriving in a new forest patch and the hazard rates of males were higher than those of females only when inter-patch distances were lower than 90 m. The opposite was observed for inter-patch distances higher than 90 m, a situation in which females have a higher hazard rate than males (Fig 5). In addition, we considered that the hazard rate was constant over time since the 95% CI of the shape parameter included the value ρ = 1 (0.93 ≤ ρ ≤ 2.25; Table 3).

**Table 3:** Model selection results for the effects of the nearest neighbor distance (NND) and sex on time spent on transfer. Parametric survival models were used in these analyses.

Rank	Model Description	- LL	k	AICc	ΔAICc	w	ρ (95% CI)
1	NND × sex	12.657	5	40.31	0	0.495	1.45 (0.93 − 2.25)
2	NND	16.481	3	40.68	0.37	0.411	1.03 (0.70 − 1.52)
3	NND + sex	16.283	4	43.64	3.33	0.094	1.04 (0.71 − 1.54)
4	Null	30.041	2	64.88	24.57	< 0.001	0.50 (0.34 − 0.73)
5	sex	29.489	3	66.69	26.38	< 0.001	0.50 (0.34 − 0.73)

– LL, negative log-likelihood; k, number of parameters; AICc, second order Akaike information criterion; ΔAICc, AICc differences; w, Akaike weight; ρ, shape parameter of Weibull distribution.

We also observed the type of locomotion during the transfer stage for eight individuals. Seven of these birds (three males and four females) hopped on the ground when crossing the matrix. We could observe only one female that flew to reach the nearest neighbor patch located 35 m from the release patch. The distance crossed by the other seven birds was higher than 80 m. In addition, the fate of the individuals that left the release patch was known for 22 birds (10 males and 12 females). Birds that did not arrive in another patch in the landscape were found predated in the matrix. We must note that the predation risk analysis was hindered because there was no convergence in the optimization of the maximum likelihood estimator of the models containing the sex predictor. In fact, only males were predated (3 out of 10 males), resulting in a lack of variability in the predation risk of females. Out of all detected predation events, we could only identify one predator (a Viperidae snake from the *Bothrops* genus). However, raptors may represent potential predators, as we frequently observed them around the areas where predation took place and we found typical traces of predation by raptors in predated animals (i.e. lots of plucked feathers accumulated in the floor, sometimes with parts of the digestive trait).

### Inter-patch movement success

**Fig 6:**
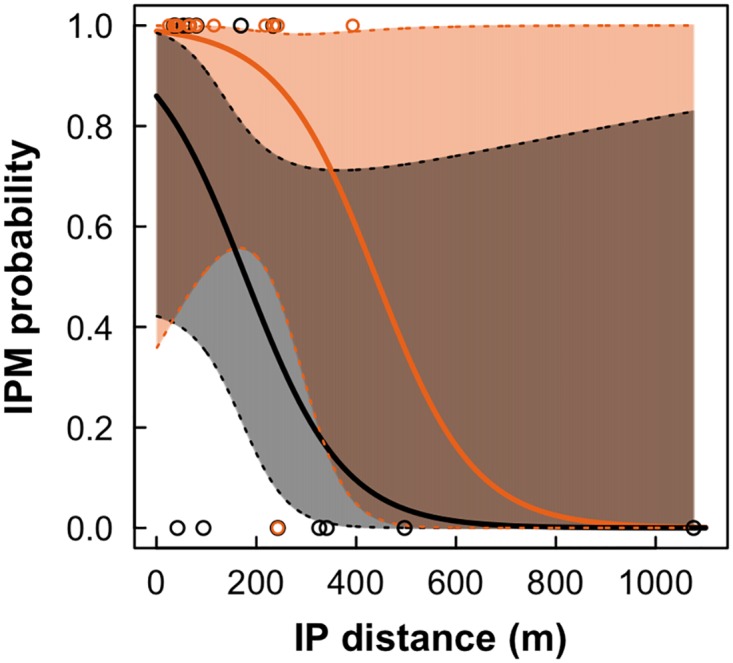
Inter-patch movement probability as a function of inter-patch distance. Curves were based on the estimated parameters of the best model, which account for the additive effects of sex and distance (β_Intercept_ = 4.46, β_DIST_ = −0.010, β_SEX(males)_ = −2.65). Black line and open circles correspond to males, and orange line and open circles to females. 95% confidence intervals were also plotted.

We obtained inter-patch movement success data for 27 birds. Twelve out of 13 translocated females (92%) were able to leave the release patch and to arrive in another patch in the landscape, whereas only seven out of 14 translocated males (50%) exhibited successful inter-patch movement. The best model to explain the inter-patch movement success of *P. leucoptera* was the model including the addictive effects of distance and sex (AICc = 26.45, w_i_ = 0.603). This model had strong support because all other models showed a ΔAICc ≥ 2.54 (Table 4). According to the estimated parameters (β_Intercept_ = 4.46, SE = 1.68; β_DIST_ = −0.010, SE = 0.005; β_SEX(males)_ = −2.65, SE = 1.39), distance had a negative effect, which was higher for males (Fig 6).

**Table 4:** Model selection results for the effects of inter-patch distance and sex on inter-patch movement success. Generalized linear models were used in these analyses.

Rank	Model Description	- LL	k	AICc	ΔAICc	w
1	NND + sex	9.70	3	26.45	0	0.603
2	NND	12.24	2	28.99	2.54	0.169
3	NND × sex	9.66	4	29.15	2.70	0.156
4	sex	13.23	2	30.96	4.51	0.063
5	Null	16.41	1	34.98	8.53	0.008

– LL, negative log-likelihood; k, number of parameters; AICc, second order Akaike information criterion; ΔAICc, AICc differences; w, Akaike weight.

## Discussion

In this study, we presented empirical evidence demonstrating that dispersal should be conceived as a three-stage process, otherwise we could not precisely describe the dispersal patterns of forest birds in fragmented landscapes neither understand the causes of these patterns. Before discussing our results, we must highlight that empirical research conceived under a three-stage framework of dispersal are lacking [1, 2, 4] and the consequences of this scarcity is dubious. On one hand, this lack makes our study pioneering in the use of the three-stage framework to empirically understand the dispersal patterns of forest birds in fragmented landscapes. On the other hand, we have no benchmarks with which we can properly compare our results and make a more general discussion. We discuss our results in relation to the few comparable studies we found and we focused also on emphasizing novel insights generated by our research in particular.

### Opening wide the limitations of a single-stage approach to study sex-biased dispersal

The sex-biased dispersal research has been mostly conducted to determine theoretical ultimate causes or unconditional behaviors explaining emigration patterns in homogeneous environments [7, 17]. Although we could not reject the classical Greenwood’s hypothesis predicting female-biased emigration for socially monogamous bird species like *P. leucoptera* [17], a higher innate emigration propensity was not sufficient to explain the female-biased immigration pattern we observed in fragmented landscapes. We discuss two interrelated reasons explaining our results, both suggesting that, in addition to a multi-stage approach, we should consider the combination of proximal (conditional) and ultimate (unconditional, innate) dispersal drivers to understand sex-biased patterns.

The first reason is that emigration was mainly affected by patch isolation (*i.e.* nearest neighbour distance). Despite any innate proneness birds may have to disperse, inter-patch distance effects suggests that some decision making process in response to the environmental condition was at stake before birds emigrate [4]. Strengthening this perspective, we were able to estimate the visual perceptual range of the study species. The fact that birds took a longer time deciding to emigrate in situations where they can not find a clear target to immigrate (i.e. neighbor patch) suggests that departure decision depends on short-term evaluations of the dispersal costs that individuals may make before effectively leaving their current patches. Assuming that all birds had the same stimulus to disperse in terms of the local conditions (*i.e.* release patch), if there was no decision making before emigration, individuals would not show different responses in relation to patch isolation. Moreover, if only an innate proneness of females drives sex-biased dispersal, the emigration proneness model including only sex as a predictor would have a higher plausibility than observed.

Castellón and Sieving was the only study we found that analysed forest birds emigration propensity [34]. These authors observed that Chucao Tapaculos, a southern temperate forest bird, were more reluctant to emigrate when the inter-patch matrix is open pastures, but emigration proneness increased when the matrix is more permeable, such as shurblands or when corridors link forest patches. However, this study did not evaluate whether there was sex-biased dispersal, hindering us to generalize the patterns we observed. Thus, it is very important that future studies deal with the problem of infering emigration costs, since conditional dispersal behaviors interact with unconditional ones (*e.g.* higher emigration proneness of females) to define dispersal patterns, as previously suggested in the literature [1]. Given that our experimental design was adapted from Castellón and Sievings’s study and despite the limitations of experimental designs involving animal translocation, we consider that Castellón and Sieving’s empirical framework is an useful and recommended approach to shed light on inferring the costs of emigration in fragmented landscapes.

The second reason we highlight to explain the relevance of a multi-stage conceptualization of dispersal is trivial: the magnitude of female-biased immigration is much higher than female-biased emigration only. We demonstrated that females had lower mortality risk and paid less costs, in terms of time, during transfer. Fahrig developed a conceptual model predicting that animal species evolving in continuous habitat landscapes had high mortality risks and costs when habitat fragmentation takes place [44]. Although we observed that this prediction was not incorrect, it is not caused by the reasons hypothesized by Fahrig, which stated that individuals from continuous habitat evolve a weak boundary response, thus a higher proneness to emigrate when the habitat becomes fragmented, even if the transfer costs are very high. If this pattern was correct, we would not expect the effect of distance or sex on emigration propensity (since Fahrig’s model do not account for individual variability, *e.g.* sex effects) and all birds would emigrate no matter the environmental conditions. So, we recommend that future studies compare the behaviors of individuals from fragmented landscapes with those from continuous forests in order to effectively test the hypotheses suggested in the Fahrig’s model.

Empirical evidence of sex-biased dispersal in birds is generally based on indirect data, such as those gathered through mark-recapture or molecular techniques. By focusing only on the immigration stage, these techniques have an important limitation as they do not cover the full dispersal process. As a consequence, we observe controversial results in the literature and the debate about sex-biased dispersal on birds remains without a consensus. On one hand, Clarke *et al.* found strong evidence of female-biased dispersal for 71 out of 102 species (70%) they studied [18]. On the other hand, Woltmann *et al.* found no evidence of female-biased dispersal for *Myrmeciza exsul*, a tropical forest Thamnophilidae as *P. leucoptera* [45]. Using molecular genetic parentage analyses these authors observed equal distributions of natal dispersal distances between males and females. Nevertheless, sample sizes were very unbalanced (12 females and 33 males) and this may be considered a relevant shortcoming/problem. Assuming that birth sex-ratio is unbiased for *M. exsul*, the unbalanced sample sizes suggests that many females were not sampled because they dispersed over longer distances than those captured in the scale of the study. Another possibility is that *M. exsul* females may have higher emigration proneness but also higher mortality risk than males, leading to a sex-unbiased immigration. Altogether, if the theoretical background underlying empirical studies on dispersal do not account for the interrelation between the three dispersal stages, inferences about sex-biased patterns and its causes will probably be mistaken.

### Transfer as a key stage to depict female-biased dispersal patterns in birds

Transfer is the stage in which the costs of moving through inhospitable and/or unknown environments are effectively paid [4], so it is a key stage of the dispersal process. Our radiotracking protocol allowed us to test some hypotheses that are longly debated in the theoretical literature on dispersal, but for which empirical validation is scarce. First, we provided empirical evidence that perceptual range is an important species attribute that should be considered when describing inter-patch dispersal patterns of forest birds in fragmented landscapes, as predicted by theoretical models (*e.g.* [13]). Second, our results showed that movement costs on transfer is strongly related with patch isolation and differ between males and females. We discuss these two interrelated topics below.

Departure direction analysis showed that the perceptual range of *P. leucotptera* is around 80–90 m, which is in accordance with the only estimate we found for understory birds (50–100 m; [46]). The inability to detect the nearest suitable patch before starting the emigration process implied in increased costs to be paid during transfer on the matrix, since individuals took random departure directions on emigration when the neareast neighbour patch is out of range. In this case, birds have a higher probability of travelling over longer routes on transfer compared to the path of the nearest neighbor patch, increasing energetic costs associated with dispersal [4]. Moreover, we observed that time costs on transfer are sex-biased. Males spent much less time than females to cross gaps shorter than 85 m, a turning point above which females spent less time than males in their movements in the matrix (Fig 5). This turning point was just above the species perceptual range, suggesting that 80–90 m is a critical distance above which *P. leucoptera*, and possibly other forest bird species of the same size, change their movement behavior.

The interactive effect of distance and sex on time spent in the matrix and the fact that birds changed their movement mode to a slower type of locomotion shed light on important aspects of forest birds dispersal in fragmented landscapes. We observed that above some threshold distance, birds tend to hop instead of flying. However, we could not determine this threshold distance, since we have just occasional observations of birds hopping and just one observation of a female flying during transfer. Despite this limitation, it is known that forest birds have low flight capability [47] and that *P. leucoptera* individuals avoid to cross gaps larger than 55 m during territorial defense movements [48]. These territorial defense movements were performed by males crossing the inter-patch matrix by flying. In this way, males may have a higher flight capability than females, spending less time to cross short inter-patch distances (*i.e.* < 85 m), while females may have greater navigation ability when dispersal distances are long and individuals move by hopping. Some factors that we were not able to control on our release sites, such as wind direction, small hedgerows or isolated trees in the matrix, may help individuals to identify cues to perform less risky movements when the environment is adverse [49–51]. Thus, once inside the matrix, females may have a greater ability to recognize non-habitat elements or paths that may provide faster and/or safer routes for dispersal. In fact, we observed females, but no males, following fencerows in cases in which the nearest neighbor distance was higher than 115 m.

An important consequence of increased time costs associated with slower movement modes in the transfer stage is that individuals experience the costs of non-linear increases on mortality risk, particularly due to death by predation [12]. The predation events we observed occurred only with males that would need to cross distances higher than 115 m to reach a suitable forest patch (see S1 Table) and, in all cases, attacked birds were hopping in an open pasture matrix. Empirical evidence of male-biased predation by raptors was previously reported for prey monitored inside their preferred habitats [52, 53]. Although our study was not designed to specifically assess the causes of sex-biased predation risk, we can suggest two hypotheses to explain these patterns, which can be evaluated in future studies. The first concerns the previously mentioned possibility that females may have a greater navigation ability than males. The second refers to the prey plumage brightness hypothesis, which states that predation vulnerability in forest passerine birds is correlated to male plumage brightness [54]. This seems a valid hypothesis here because in a brownish-green background (i.e. a pasture and cropland matrix), black males may appear brighter than brownish females to visual predators, such as raptors.

Altogether, our study provided empirical evidence supporting the hypothesis that sex-biased dispersal in fragmented landscapes is driven by a higher emigration propensity and a lower mortality risk on transfer for females. Nevertheless, generalization of our results must be done with caution due to the scarcity of empirical studies providing us a benchmark for comparisons. We could only find two relevant studies explicitly addressed dispersal as a three-stage process [55, 56]. In a microscale laboratory system, Bowler and Benton [55] investigated how food availability for juvenile soil mites affected their dispersal movements when individuals become adult. They observed that in the low food availability treatment, which is the closest scenario in relation to our framework, there was no evidence for sex-biased dispersal. However, in the case of high food availability, males were more prone to emigrate, had a lower mortality on transfer and higher immigration success than females. This is the same pattern we observed, but in the opposite direction. In a mesoscale experimental system, Trochet and collaborators [56] investigated how the sex-ratio in a habitat patch affected the dispersal movements of the butterfly species *Pieris brasicae*. These authors did not find statistically significant evidence of a sex-biased dispersal pattern, except for the male-biased sex-ratio treatment, in which only the immigration stage was male-biased. Summing up, these results just confirmed that the dispersal pattern of an individual is a function of the proximate motivations eliciting these movement process (e.g. resource availability or sex ratio) and vary between species or higher taxonomic groups (e.g. mites, insects and birds). Our contribution was to empirically demonstrate in macroscale field experiments that species interactions such as predation play a crucial role in defining the (sex-biased) dispersal pattern of a tropical forest bird.

### Theoretical and applied consequences of female-biased dispersal in fragmented landscapes

We presented strong evidence of a female-biased dispersal pattern for *P. leucoptera*, but we are still far from elucidating the effects of sex-biased dispersal on population dynamics in fragmented landscapes [57]. Based on a literature review, Dale hypothesized that female-biased dispersal may increase the risk of extinction when populations are small and isolated [57]. He predicted population declines as a consequence of male-biased sex ratio, thus low reproductive success on small patches when emigration is female-biased. However, empirical evidence corroborating Dale’s hypothesis are lacking as well as quantitative models predicting demographic or metapopulational consequences of female-biased dispersal and male-biased sex ratio in small patches [58, 59]. Modeling population dynamics in space and time and considering individual variability has been challenging ecologists for a long time [60]. Individual-based models (IBMs) have been considered as an important approach to overcome this difficulty [2, 61]. For example, Fahrig developed a simple IBM to understand how reproductive rate, mortality in the matrix, habitat configuration and movement rates affect the extinction threshold of a species [62]. If Dale’s predictions are correct, extinction thresholds may be higher than expected by Fahrig’s model. In this way, we recommend that extensions of Fahrig’s model considering different dispersal patterns between males and females should be developed and empirically validated.

Our study also has implications for the management of fragmented landscapes. We demonstrated that dispersal is constrained by patch isolation, thus we expect low habitat connectivity in highly fragmented landscapes, such as the one studied here. Habitat connectivity is pivotal to determine the amount of available habitat for an individual [63], which, in turn, is an important predictor of *P. leucoptera*’s incidence in fragmented landscapes [48]. Linking habitat patches with forest corridors became a widely suggested management procedure to restore habitat connectivity for forest birds, since the conduit role of these corridors has been recently demonstrated (e.g. [34, 64, 65]). As our findings indicates, the effectiveness of forest corridors for the conservation of *P. leucoptera* will depend on whether this structures can provide an increased stimulus to male emigration, as well as on whether corridors reduce the risk of male predation. Another possibility to increase landscape connectivity is to implement stepping stones in the matrix. Although stepping stones may facilitate inter-patch foraging movements [49], applying this management procedure in highly fragmented landscapes must be taken with caution. Stepping stones effectiveness may decrease severely when inter-patch distances are long due to deferred costs [66] or even for not providing a sufficient stimulus and protection for males. Therefore, future studies demonstrating the usefulness of forest corridors or stepping stones not only for *P. leucoptera*, but also for other forest birds are recommended.

### Conclusion

Dispersal research is passing through a period of intense conceptual reorganization and the problems of conceiving dispersal as a single stage process or focusing only on unconditional mechanisms driving dispersal patterns have been systematically pointed out in the literature. Following the novel trends on dispersal research, we found strong evidence supporting that we should move on from simplistic assumptions to study dispersal phenomena. Methodological constrains remains an issue hindering the elaboration and implementation of proper experimental designs to obtain the necessary empirical evidence to test many hypotheses concerning dispersal. However, recent technological advances on radiotransmitters development is allowing ecologists to overcome many limitations that used to preclude empirical research. Our study demonstrates how an experimental design involving translocation and radiotracking, as well as, an analytical approach interrelating the three stages of dispersal can be used to shed light on the causes and consequences of important dispersal patterns, such as the female-biased emigration, transfer and immigration of forest birds in fragmented landscapes. This framework should be easily adapted to study other dispersal problems and hypotheses (*e.g.* the effects of seasonality, age, other matrix types or the presence of conspecifics in neighboring patches). In conclusion, we emphasize the use of experimental and analytical frameworks allowing an integrated understanding of the dispersal process, aiming to merge both evolutionary and ecological knowledge, as well as to depict the influences of ultimate and proximate behaviors on dispersal patterns. Empirical studies in this direction will certainly generate important insights not only on elucidating dispersal patterns themselves but also on predicting population dynamics consequences with more precision and elaborating proper management procedures to mitigate the negative impacts of forest fragmentation.

## Supporting Information

S1 Fig Study Region. (A) Location of the study area on the Atlantic Plateau of the State of São Paulo, Brazil and (B) a detailed view of this area, highlighting the location of the experimental landscapes (black circumferences). In (B), forest patches are represented in dark grey, and continuous forest was located inside the hatched area.
(TIF)

S2 Fig Hazard rates for emigration propensity. Variation of hazard rates in relation to the nearest neighbour distance for birds remaining for at least 12, 24, 60, 120, and 180 daylight hours in the release patches. The red curve highlights the variation of the hazard rates for birds remaining at least the time equivalent to our systematic sampling protocol (i.e. 60 daylight hours curve).
(TIF)

S1 Table Raw data. This table provides a summary of release site spatial characteristics and the raw dataset analyzed in main text (including explanatory and response variables). Nearest neighbor distance, NND; Shortest path distance, SPD. Survival times are given in daylight hours and Survival times (matrix) in hours. The Emigration, Predation and Immigration columns refers to the occurrence or not of these events (0 = not occurred; 1 = occurred).
(PDF)
