# Supplementary figures and images for: High Emigration Propensity and Low Mortality on Transfer Drives Female-Biased Dispersal of Pyriglena leucoptera in Fragmented Landscapes
Source: PLoS One. 2017 Jan 20;12(1):e0170493. doi: 10.1371/journal.pone.0170493 (PMC5249090; doi:10.1371/journal.pone.0170493)

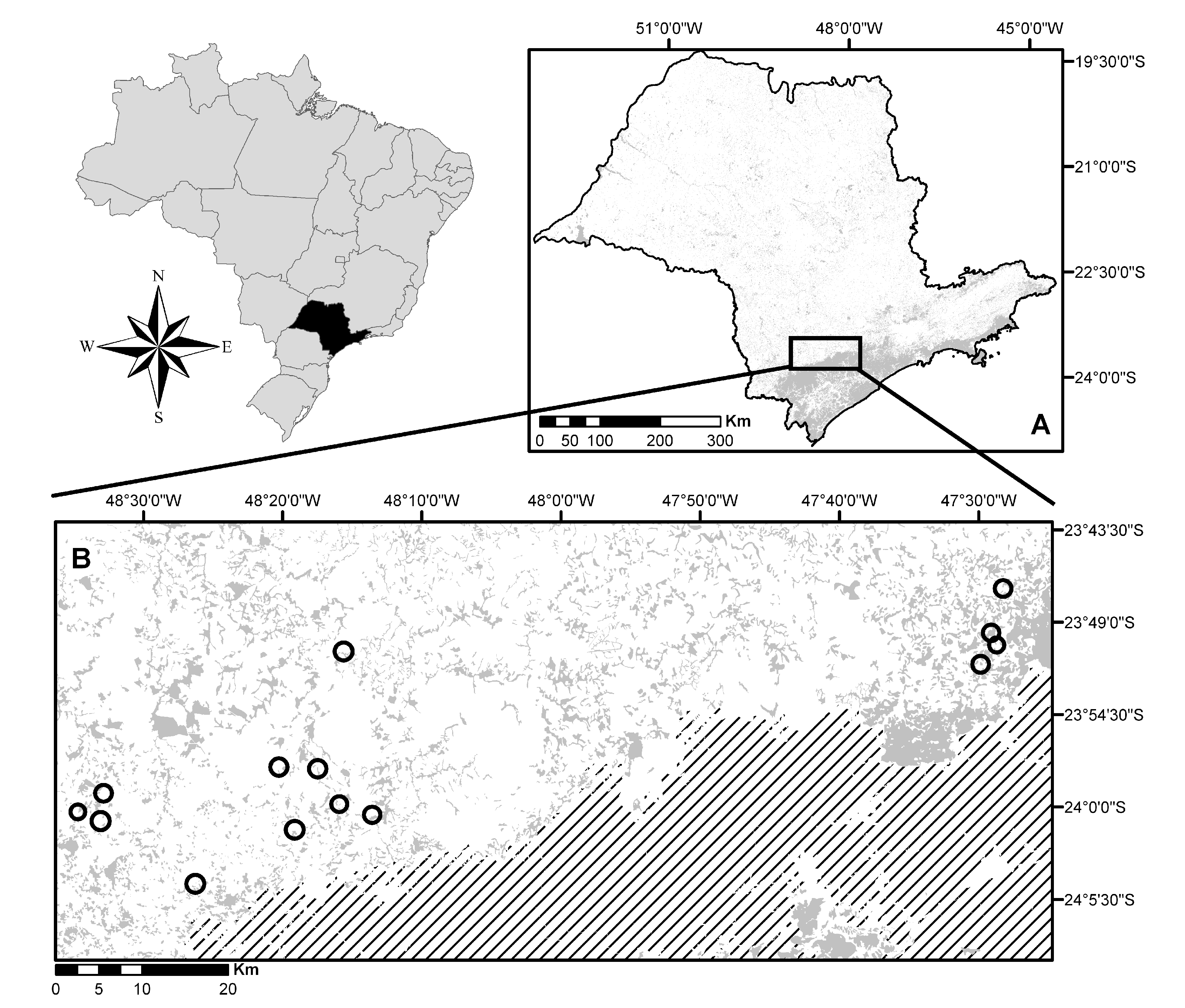

Supplement: S1 Fig — (A) Location of the study area on the Atlantic Plateau of the State of São Paulo, Brazil and (B) a detailed view of this area, highlighting the location of the experimental landscapes (black circumferences). In (B), forest patches are represented in dark grey, and continuous forest was located inside the hatched area. (TIF) [file pone.0170493.s001.tif]

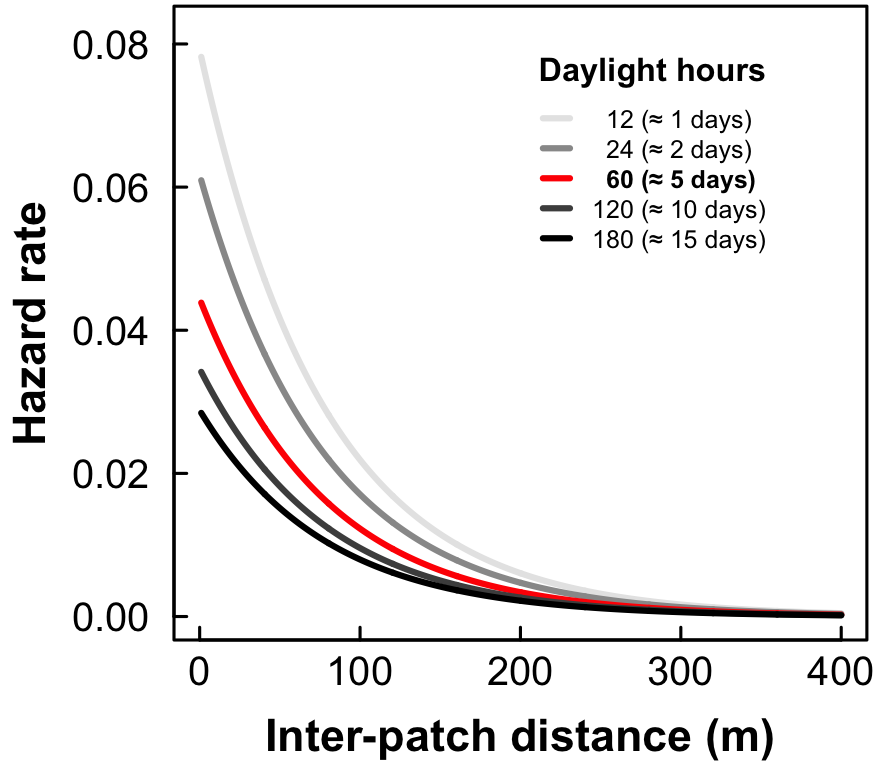

Supplement: S2 Fig — Variation of hazard rates in relation to the nearest neighbour distance for birds remaining for at least 12, 24, 60, 120, and 180 daylight hours in the release patches. The red curve highlights the variation of the hazard rates for birds remaining at least the time equivalent to our systematic sampling protocol (i.e. 60 daylight hours curve). (TIF) [file pone.0170493.s002.tif]
