# Supplementary material for: High Emigration Propensity and Low Mortality on Transfer Drives Female-Biased Dispersal of Pyriglena leucoptera in Fragmented Landscapes
Source: PLoS One. 2017 Jan 20;12(1):e0170493. doi: 10.1371/journal.pone.0170493 (PMC5249090; doi:10.1371/journal.pone.0170493)
Supplement: S1 Table — This table provides a summary of release site spatial characteristics and the raw dataset analyzed in main text (including explanatory and response variables). Nearest neighbor distance, NND; Shortest path distance, SPD. Survival times are given in daylight hours and Survival times (matrix) in hours. The Emigration, Predation and Immigration columns refers to the occurrence or not of these events (0 = not occurred; 1 = occurred). (PDF) [file pone.0170493.s003.pdf]

Release Site (RS) Spatial Descriptors					Other Explanatory Variables		Response Variables					
Code	Tested Season	Release Patch Size (ha)	Nearest Neighbor Size (ha)	NND (m)	DDD (m)	Sex	Survival times	Emigration	Departure Direction (degrees)	Survival times (Matrix)	Predation	Imigration*
RS1	Wet	0.19	33.69	94	94	M	72.32	0	NA	NA	NA	0
RS1	Wet	0.19	33.69	94	243	F	56.75	1	263	1.63	0	1
RS2	Dry	0.92	20.72	35	35	M	6.23	1	39	0.05	0	1
RS2	Dry	0.92	20.72	35	35	F	12.58	1	1	0.02	0	1
RS3	Wet	0.28	118.77	65	65	M	9.75	1	4	0.50	0	1
RS3	Wet	0.28	118.77	65	65	F	6.47	1	355	0.50	0	1
RS4	Dry	1.38	27.68	54	54	M	4.17	1	356	0.50	0	1
RS4	Dry	1.38	27.68	54	67	F	9.93	1	NA	NA	NA	1
RS5	Dry	0.55	4.30	139	234	F	0.50	1	57	22.82	0	1
RS5	Dry	0.55	4.30	139	234	M	2.50	1	NA	NA	NA	1
RS6	Dry	3.31	91.70	243	243	F	54.88	0	NA	NA	NA	0
RS6	Dry	3.31	91.70	243	243	M	153.85	0	NA	NA	NA	0
RS7	Wet	2.00	45.63	80	80	F	1.00	1	4	0.50	0	1
RS7	Wet	2.00	45.63	80	80	M	4.17	1	9	0.50	0	1
RS8	Dry	1.21	17.79	25	1076	M	0.50	1	224	15.07	1	0
RS8	Dry	1.21	17.79	25	25	F	4.42	1	325	0.50	0	1
RS9	Wet	1.59	47.69	42	42	F	25.25	1	346	1.00	0	1
RS9	Wet	1.59	47.69	42	42	M	87.12	0	NA	NA	0	0
RS10	Wet	2.66	51.02	165	326	M	13.97	1	238	2.95	1	0
RS10	Wet	2.66	51.02	165	165	F	14.28	0	NA	NA	NA	NA
RS11	Dry	0.59	70.10	100	218	F	13.40	1	198	NA	0	1
RS11	Dry	0.59	70.10	100	169	M	0.75	1	62	0.25	0	1
RS12	Wet	0.65	3.50	341	341	M	203.02	0	NA	NA	NA	0
RS12	Wet	0.65	3.50	341	393	F	1.77	1	238	25.47	0	1
RS13	Wet	0.44	6.01	115	115	F	12.03	1	NA	NA	NA	1
RS13	Wet	0.44	6.01	115	497	M	1.08	1	120	2.50	1	0
RS14	Wet	1.27	53.29	38	38	F	5.80	1	358	0.50	0	1
RS14	Wet	1.27	53.29	38	38	M	11.37	1	333	0.50	0	1
RS13	Wet	0.44	6.01	115	497	F	12.78	1	115	NA	NA	NA
RS10	Wet	2.66	51.02	165	165	M	10.72	0	NA	NA	NA	NA
RS 6	Dry	3.31	91.70	243	243	F	18.52	0	NA	NA	NA	NA
